# A Review of Selective Laser Melted NiTi Shape Memory Alloy

**DOI:** 10.3390/ma11040519

**Published:** 2018-03-29

**Authors:** Zhong Xun Khoo, Yong Liu, Jia An, Chee Kai Chua, Yu Fang Shen, Che Nan Kuo

**Affiliations:** 1 Institute for Sports Research, School of Mechanical and Aerospace Engineering, Nanyang Technological University, Singapore 639798, Singapore; zkhoo001@e.ntu.edu.sg; 2 School of Mechanical and Aerospace Engineering, Nanyang Technological University, Singapore 639798, Singapore; yliu.email@yahoo.com; 3 Singapore Centre for 3D Printing, School of Mechanical and Aerospace Engineering, Nanyang Technological University, Singapore 639798, Singapore; anjia@ntu.edu.sg (J.A.); mckchua@ntu.edu.sg (C.K.C.); 4 Department of Bioinformatics and Medical Engineering, Asia University, Taichung 41354, Taiwan; cherryuf@gmail.com; 5 3D Printing Medical Research Institute, Asia University, Taichung 41354, Taiwan

**Keywords:** 3D printing, 4D printing, additive manufacturing, Selective Laser Melting, NiTi, shape memory alloy

## Abstract

NiTi shape memory alloys (SMAs) have the best combination of properties among the different SMAs. However, the limitations of conventional manufacturing processes and the poor manufacturability of NiTi have critically limited its full potential applicability. Thus, additive manufacturing, commonly known as 3D printing, has the potential to be a solution in fabricating complex NiTi smart structures. Recently, a number of studies on Selective Laser Melting (SLM) of NiTi were conducted to explore the various aspects of SLM-produced NiTi. Compared to producing conventional metals through the SLM process, the fabrication of NiTi SMA is much more challenging. Not only do the produced parts require a high density that leads to good mechanical properties, strict composition control is needed as well for the SLM NiTi to possess suitable phase transformation characteristics. Additionally, obtaining a good shape memory effect from the SLM NiTi samples is another challenging task that requires further understanding. This paper presents the results of the effects of energy density and SLM process parameters on the properties of SLM NiTi. Its shape memory properties and potential applications were then reviewed and discussed.

## 1. Introduction

Shape memory alloy (SMA) is one category of smart material that has the capability to convert thermal energy into mechanical work [1,2]. It is commonly used in special engineering applications due to its high actuation energy densities and exceptional functional properties [3,4,5,6]. Among the different SMAs, nickel titanium (NiTi) has the best shape memory behavior including high magnitudes of shape recovery, recovery stress, and superelastic strain [3,7]. As a result, NiTi can be found in various fields of application such as actuators [8,9,10], aerospace [3,11], biomedical implants and fixtures [3,5,8,9,11,12,13,14,15,16,17], couplings and fasteners [10], electrical safety devices [8], micro-electromechanical systems (MEMS) [8,18], naval [3], smart composites [19], sporting equipment [3], surgical instruments [3], etc. 

The NiTi SMA possesses both the shape memory effect (SME) (thermal memory) and superelasticity (mechanical memory) [4,6,12,20]. To fully utilize these properties, NiTi needs to be processed into various geometries for different applications. However, conventional manufacturing methods have several limitations including the contamination of the crucible by oxygen [13]. In addition, NiTi is a material that is difficult to process due to its compositional sensitivity and poor machinability [5,14,20,21,22,23]. Thus, most of the conventionally-produced NiTi parts have simple geometries such as in the form of wires, plates, bars, tubes, etc., which has critically limited the full potential applicability of NiTi [5,20,21,22]. Therefore, in recent years, additive manufacturing or 3D printing techniques such as Selective Laser Melting (SLM) have been proposed as a solution to produce net-shape complex NiTi structures [4,6,12,13,14,15,16,18,20,21,24,25,26,27,28,29,30,31,32]. This new fabrication method is commonly known as 4D printing. 

4D printing has been defined as an additive manufacturing process that incorporates smart materials as the raw materials needed for the fabrication of 3D structures [30]. These produced components are able to respond in an anticipated manner to external stimuli from the environment or through human intervention, leading to a change in shape or physical properties over time. In the research regarding 4D printing, 3D printed shape memory polymers have been extensively reported [33,34,35,36]. Some studies have even dived into the reversibility of 3D printed shape memory materials [37]. However, little work has been reported on SLM-produced NiTi with comparable properties to the conventional NiTi. Moreover, different SLM equipment would most probably require different combinations of optimized process parameters due to various factors; the type of laser used, powder properties, chamber conditions, etc. No clear guideline has been established yet on obtaining SLM NiTi parts with the preferred transformation characteristics and properties. Furthermore, the SLM parameters and the resultant energy density (a function of various parameters) have strong impacts on the properties of the samples manufactured. This paper reviews some of the results of the effects of energy density and process parameters on the properties of SLM-produced NiTi, their shape memory responses, and potential applications. More emphasis was placed on repetitive laser scanning as an alternative approach to the production of high-quality samples [31]. These repetitive-scanned samples were subjected to SME testing under tensile mode. The tensile test is an essential test to determine the overall quality of SLM NiTi including their porosities, transformation characteristics, and SME. The majority of the shape memory results reported were conducted under a compression load, which neglected the effects of porosity. The ability of the repetitive-scanned SLM NiTi samples to withstand tensile loads up to 8% with the highest shape recovery of 5.11% demonstrated good mechanical properties, negligible deviation in chemical composition, and decent shape memory effect. Finally, through this review, it is hoped to reduce the time and resources needed for the determination of optimized parameters for future research.

## 2. SLM of NiTi SMA

The SLM process has the advantage of fabricating complex structures in a layer-by-layer fashion without the need for machining [38]. Thus, it presents a new manufacturing route that addresses the poor machinability of NiTi. Furthermore, due to the endless possibility of producing SLM NiTi for both existing and novel applications, many studies have been conducted and reported in this research field [4,6,12,13,14,15,16,18,20,21,24,25,26,27,28,29,30,31,32]. Nevertheless, different fabrication conditions influence the properties of SLM NiTi differently.

Studies have shown that similar magnitudes of energy density do not always produce samples, be it conventional metals or NiTi SMA, with comparable or identical properties [6,39,40]. Different phenomena on the effects of energy density [12,16,20,21] and SLM process parameters [6,16,24,27] on the mechanical properties, phase transformation characteristics, and shape memory responses of NiTi have been reported. Providing non-optimal energy density and/or using inappropriate parameters will result in samples produced with issues such as high porosity, balling effect, warpage, deviated chemical composition, etc. One common problem encountered in the fabrication of SLM NiTi is the depletion of Ni [16,21,26], possibly leading to large deviations in the transformation temperatures [16]. In this section, the influences of energy density and SLM process parameters on the properties of produced SLM NiTi are reviewed and discussed. Next, their shape memory properties and potential applications are also presented. 

### 2.1. Effects of Energy Density

In general, the density of SLM-produced parts increases with increasing energy density directed to the powder bed [18]. The magnitude of energy density delivered is usually calculated using Equation (1) [12,16,32,39,41,42,43,44,45].

(1) $$E=\frac{P}{vhd}$$

where *E* refers to the energy density (J/mm^3^); *P* denotes the laser power (W); *v* indicates the scanning speed (mm/s); *h* signifies the hatch distance (mm); and *d* represents the layer thickness (mm).

Among the different research conducted, Meier et al. [21] and Haberland et al. [20] reported that the minimum energy density required to produce a highly dense SLM NiTi sample was 85 and 200 J/mm^3^, respectively. Nonetheless, three adverse effects were observed when the energy density increased beyond the optimal magnitude.

First, Bormann et al. [16] and Haberland et al. [20] noticed an increase in the porosity of their samples with increasing energy density above the critical amount, leading to a decrease in the relative density by up to about 10%. There are two possible explanations for this phenomenon. First, it could have been due to the increase in the volume of the molten pool [16]. Thus, the diffusion of the gas bubbles to the surface during the solidification process was hindered, resulting in the production of high porosity samples. Second, an excessive amount of energy density could have led to the balling effect and the formation of voids [20]. Eventually, the fabricated samples exhibited high porosity. Nevertheless, Meier et al. reported a contradicting result [21] where they observed a minimal effect on the density of the produced parts as energy density continued to increase above the minimum value needed.

Second, it has been reported that the transformation temperatures of the SLM NiTi samples increase as the energy density delivered increases [12,20]. The increase could be as much as 75 °C [12]. The occurrence of this phenomenon is the result of Ni loss via evaporation as Ni has a lower evaporation temperature than Ti (evaporation temperature of Ni is 3186.15 K and the evaporation temperature of Ti is 3560.15 K) [12,16,20,46,47]. Furthermore, the increase in the transformation temperatures coincides with the results of other researchers who have also detected a drop in Ni content in their SLM NiTi samples [16,21,26].

Third, Haberland et al. observed a direct relationship between the impurity-pick up by SLM NiTi and the input energy density magnitude [20]. In their study, they adopted an inert environment for the fabrication of their SLM NiTi samples. Nevertheless, the amount of oxygen and nitrogen picked up by their samples increased significantly by up to about 0.14 wt % as the energy density delivered continued to increase above the minimum magnitude. This was most likely due to the material experiencing a higher temperature, larger dimension of the molten pool, and a lower rate of solidification when exposed to unnecessarily high energy density. Collectively, these three reported adverse effects illustrate the importance of determining the optimal energy density required. This is to produce a SLM NiTi part that exhibits high density, desired transformation temperatures, and low impurity content.

### 2.2. Effects of Process Parameters

The studies mentioned in the previous section presented the potential for controlling the chemical composition and transformation temperatures of SLM NiTi via the alteration of energy density. On the other hand, Dadbakhsh et al. [6] and Speirs et al. [26] demonstrated the possibility of influencing the transformation characteristics by varying the different SLM process parameters.

In the research by Dadbakhsh et al., they came up with various combinations of parameters with similar magnitudes of energy density [6]. These sets of parameters were categorized as low laser parameters (LP) and high laser parameters (HP). LP was associated with low laser power (40 W), low laser scanning speed (160 mm/s), and lower heating and cooling rates. HP corresponded to high laser power (250 W), high laser scanning speed (1100 mm/s), and higher heating and cooling rates. These two combinations of parameters were capable of producing SLM NiTi samples with comparable chemical composition and approximately 99% density. 

However, the samples produced by the different parameter combinations demonstrated different transformation temperatures. Samples that were fabricated using the LP combination exhibited the martensitic phase and SME while samples produced with the HP combination exhibited the austenitic phase and superelasticity at room temperature. The difference in the transformation temperatures was attributed to the different cooling rates, grain sizes, and precipitations [6].

Dadbakhsh et al. proposed the formation of precipitations as a possible reason for the higher transformation temperatures of the LP samples [6]. The presence of the precipitates generated incoherency stresses and altered the local Ni content of the Ni-rich NiTi. As the scanning speed used for the LP combination was lower, more time was available for growing the precipitates. Thus, the formation of these precipitates led to the increase in the transformation temperatures of the LP samples. 

Additionally, Meier et al. also reported the presence of Ti_2_Ni precipitates in their SLM NiTi samples [21]. These precipitates formed during the solidification process. As a result of the geometric conditions, heat transfer during the SLM process was not uniform. Hence, the regions with reduced heat transfer experienced a higher temperature for a longer period. Eventually, more Ni was lost due to evaporation and ultimately led to the formation of Ti_2_Ni precipitates. Meanwhile, any content variation of NiTi caused the transformation temperatures to change significantly. In this case, the presence of Ti-rich NiTi due to the evaporation of Ni led to the increase of the transformation temperatures. As expected, this coincided with what Meier et al. observed in their SLM NiTi samples [21]. 

On the other hand, since the HP combination produces a higher cooling rate, it may have resulted in the presence of higher Ni content and less precipitation due to the reduction in Ni evaporation. Moreover, the smaller grain size due to the higher cooling rate could also have caused a significant decrease in the transformation temperatures. Thus, the HP combination tends to result in the lowering of the austenitic transformation start and finish (A_s_ and A_f_) temperatures of the SLM NiTi samples [6]. 

Based on these results, Dadbakhsh et al. continued to explore the effects of SLM parameters on the mechanical behavior and geometrical characteristics of porous scaffold components fabricated with HP and LP combinations [15]. Such porous structures can provide a suitable environment for cell seeding and vascularization and still act as a load bearing medium concurrently [14,15]. 

According to their findings, the HP combination has resulted in a large geometrical mismatch between the actual produced thin struts and the computer-aided design (CAD) model. The main reason was that of the longer time required to accelerate and decelerate the Galvano scanner to achieve a higher scanning speed. Instead, a lower scanning speed was obtained during the scanning process of producing thin struts. Consequently, the energy density directed to the powder particles would be higher than desired, resulting in the formation of larger molten pools and thicker struts. 

Moreover, the HP samples were observed to fracture at a higher compressive load than the LP samples. This was mainly due to the thicker struts formed as they provide a more uniform distribution of strains. In addition, the HP samples also demonstrated a better load bearing ability against directional loading. Besides the geometrical differences, the higher cooling rate experienced by the HP samples could have also led to a finer microstructure and higher overall strength. One suggestion to achieve higher scanning speed would be to adopt point scanning to avoid the acceleration and deceleration of the Galvano scanner [15]. 

In a separate study performed by Speirs et al., they reported that the oxygen content present during the fabrication process had a strong effect on the transformation characteristics of the SLM NiTi samples [26]. Although an identical magnitude of energy density was delivered, samples produced in a low-oxygen atmosphere (about 220 ppm oxygen) had comparable transformation temperatures. A huge difference in transformation temperatures existed for the samples fabricated under a high-oxygen environment (about 1800 ppm oxygen). Furthermore, high-oxygen content resulted in the formation of brittle oxides and led to harmful mechanical responses. These results suggested that the quality of the produced SLM NiTi parts was heavily dependent on the atmospheric condition. 

Other than affecting the transformation characteristics, SLM process parameters can also have an effect on the porosity of the samples produced. Bormann et al. observed a decreasing trend in the relative density of their SLM NiTi samples with an increase in the exposure time (point distance divided by laser scanning speed) [16]. The occurrence of these two phenomena: (1) a decrease in relative density by up to about 10% as energy density increases above optimal value; and (2) a decrease in relative density by up to roughly 7% as scanning speed decreases or as exposure time increases, can be associated to the volume of the molten pool [16]. The amount of NiTi powder melted and the volume of molten pool formed is expected to increase as input energy density rises or as scanning speed decreases. Hence, the diffusion of the gas bubbles within the molten pool to the surface will be progressively hindered during the process of solidification. Subsequently, it causes a larger number of closed pores and the production of high porosity samples. 

Generally, the results reported by Dadbakhsh et al., Speirs et al., and Bormann et al. implied a strong influence of SLM process parameters on the transformation characteristics and porosity of SLM NiTi. This interesting discovery has demonstrated another possibility of obtaining the desired transformation temperatures by varying the process parameters in addition to energy density alone. 

Nevertheless, the presented findings on the effects of energy density and SLM process parameters did not offer a clear answer to the complicated problems encountered in the study of SLM NiTi. This is a result of the dependency of energy density on the laser power, laser scanning speed, hatch distance, and powder layer thickness as shown in Equation (1). Varying the parameters will alter the magnitude of the energy density inputted. Therefore, the effects of energy density and SLM parameters cannot be well differentiated as it is impossible to have only one variable. The adjustment of energy density will result in the variation of another parameter and vice versa. This may also be the reason why various researchers have reported different phenomena. For instance, Zhang et al. stated that the laser scanning speed did not have much effect on the transformation temperatures of SLM NiTi [24]. However, this contradicts the work reported by Bormann et al. [16] and Speirs et al. [26]. Bormann et al. observed that the amount of Ni lost decreased with increasing exposure time or reduction in scanning speed, indicating that Ni evaporation was time-dependent [16]. They proposed the formation of oxide on the surface of the molten pool as the reason for obstructing the amount of Ni evaporated. Speirs et al. noted a decrease in the martensitic transformation start (M_s_) temperature as the scanning speed increased [26]. Unquestionably, more studies are required to validate these contradictory findings. Even though it is possible to have constant energy density while the scanning speed varies, the other parameters also have to be altered in order to achieve that. Therefore, the results reported on the effects of scanning speed at a given constant energy density may not be totally accurate. 

**Figure 1:**
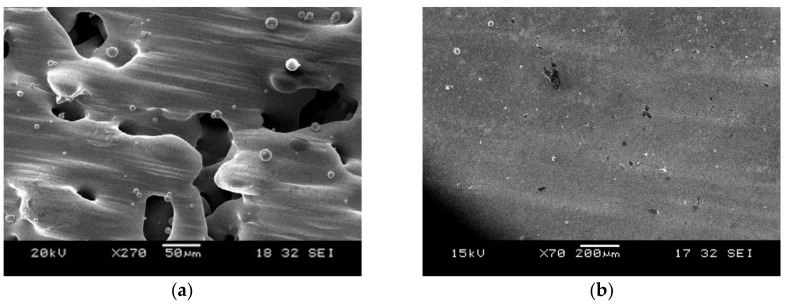
SEM images of a sample produced using laser power of (**a**) 25 W during S1, where it illustrates the extent of melting of the powder particles, followed by (**b**) 60 W during S2, where it shows that there is no obvious unmelted powder or pore inside of the optimized sample after the second scan.

In our research of fabricating high-quality SLM NiTi, we introduced another process called repetitive scanning [31]. The samples were produced by two-time scanning with different laser power for each scan. A laser power of 25 W was adopted for the first scan (S1), followed by a laser power of 60 W during the second scan (S2). The laser scanning speed was fixed at 3.6 m/s. Four square samples of 5 mm × 5 mm were fabricated for the study of their surface morphologies using scanning electron microscopy (SEM). The SEM images of the sample produced after S1 and S2 are shown in Figure 1.

Without exhibiting warpage, porosity, and balling effect, Figure 1b illustrates that the samples demonstrated flatter and smoother surfaces after repetitive scanning. Thus, four long strip samples of dimension 3.5 mm × 78 mm were further fabricated to test their transformation strains. The results were very appealing and will be discussed in the next section.

### 2.3. Shape Memory Responses of SLM NiTi

Other than influencing porosity and transformation characteristics, the SLM process was also found to strongly orient the austenitic crystals towards the building direction [27]. Another work by Dadbakhsh et al. demonstrated the effects of austenitic crystals orientation on the shape memory responses of SLM NiTi [27]. It was discovered that the samples aligned vertically to the building stage exhibited the highest percentage of elastic recovery upon removal of the compressive force. However, these samples had the lowest transformation strain measured. On the other hand, samples that were produced horizontally to the building stage had the lowest elastic recovery and highest shape recovery strain upon heating. A suggested reason for the difference in shape memory responses is due to the reduction in the stability of twinned martensite formed as a result of the elongated austenite crystals. 

Furthermore, the orientation of the austenite sub-grains was also observed to influence the mechanical properties of the produced samples [27]. The material had a higher resistance to the compressive load applied when the samples were produced horizontally. The resistance decreased for samples fabricated in a vertical manner. This occurrence was suggested to be associated with the more ordered arrangement of the martensite twins when the compressive force was applied horizontally.

In addition, Dadbakhsh et al. proposed that the internal stresses introduced during the SLM process may have induced the formation of large martensitic plates [27]. These plates would lead to a decrease in the elastic recovery of the samples upon unloading. Although annealing followed by furnace cooling could remove these martensitic plates, these treatments could not produce isotropic properties in the samples. Introducing furnace cooling after annealing may even have resulted in the segregation of austenite and martensite within the solidified scanned track, leading to a mixed shape memory response. 

In another separate study, Haberland et al. demonstrated the potential in producing high-quality SLM NiTi samples that exhibited SME and superelasticity upon compression loading [20]. They compared their samples to the conventionally-produced NiTi with identical chemical composition. Both the near-equiatomic SLM NiTi and conventional NiTi samples exhibited the typical cyclic behavior. For each cycle, some irreversible deformation and shape recovery via SME were observed. The amount of irreversible deformation accumulated during the cycling testing, while showing a decreasing trend as the number of cycles increased. However, the magnitude of residual strain differed for both samples. The conventional NiTi had a higher residual strain in the first few cycles while the SLM NiTi had a higher residual strain in the later cycles. Nonetheless, the irreversible effects were less pronounced for the SLM samples. Furthermore, the decrease in the maximum strain of each cycle was more prominent for the conventional NiTi. Hence, these observations implied that the SLM-produced samples had a more stable shape recovery. Moreover, the comparison of the ratio of reversible strain (sum of strains recovered upon unloading and upon heating via the SME to the maximum strain) developed for each cycle showed that the SLM NiTi samples had a more stable and higher shape recovery in the initial cycles. No difference was observed between the SLM and conventional samples after the sixth cycles. 

In the case of the superelasticity behavior, it was evaluated from the Ni-rich conventional and SLM samples [20]. Both materials illustrated superelasticity with the typical mechanical hysteresis loop. For each testing cycle, these materials exhibited superelastic shape recovery with some irreversible deformation. Likewise, the amount of irreversible deformation accumulated as the number of cycles increased, with an additional decrease in the hysteresis width with each cycle. Additionally, the maximum and reversible strains decreased as the number of cycles increased. Nevertheless, the SLM NiTi samples still appeared to demonstrate a higher maximum strain and slightly higher amount of shape recovery than the conventional NiTi.

Moreover, the NiTi parts produced by SLM can also exhibit different extents of shape recovery per unit change in temperature. In the research done by Meier et al. [4,21], Bormann et al. [12], Dadbakhsh et al. [6,15,27], and Haberland et al. [20], sharp transformation peaks were observed during the differential scanning calorimetry (DSC) tests. However, Clare et al. managed to fabricate SLM NiTi samples with a much gradual phase transformation than the conventional NiTi [18]. The potential applications related to the gradual phase change will be discussed in the next section. 

**Figure 2:**
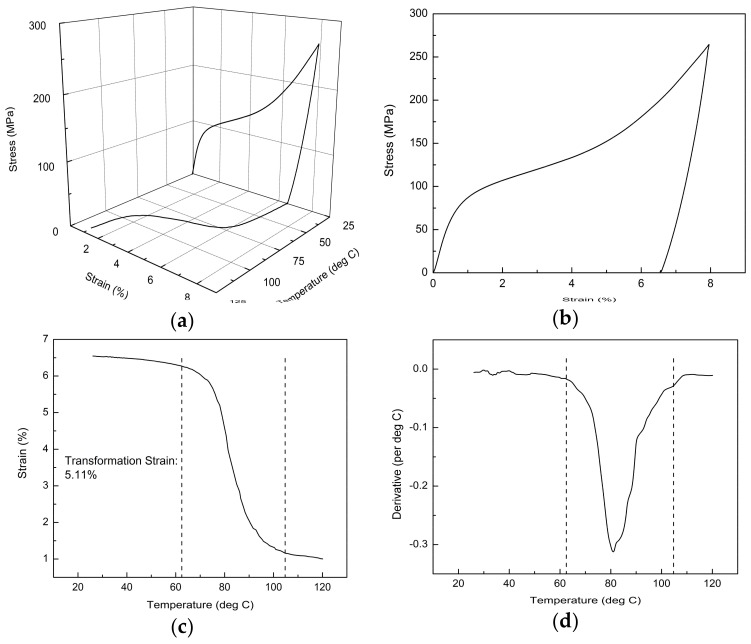
(**a**) Stress-strain-temperature curve of the best sample tested. The respective (**b**) stress-strain curve, (**c**) strain-temperature, and (**d**) its derivative curve are presented.

As described in the previous section, the introduction of repetitive scanning has produced SLM NiTi samples with seemingly excellent properties. Hence, the samples were subjected to further testing to determine the magnitude of transformation strain. Furthermore, a minimal number of tensile tests have been reported although most of the shape memory tests for SLM NiTi were conducted under compression [4,6,15,20,27]. Nonetheless, the problem with the compression test is that it did not consider the effect of porosity. Therefore, in determining the overall quality of SLM NiTi including porosities, transformation characteristics, and SME, tensile testing is essential. Figure 2 presents the thermomechanical behavior of the best sample tested under tensile mode. The derivative curve was used to obtain the temperatures that corresponded to the start and end of shape recovery. Table 1 presents the various strain readings of the same sample tested in Figure 2.

**Figure 3:**
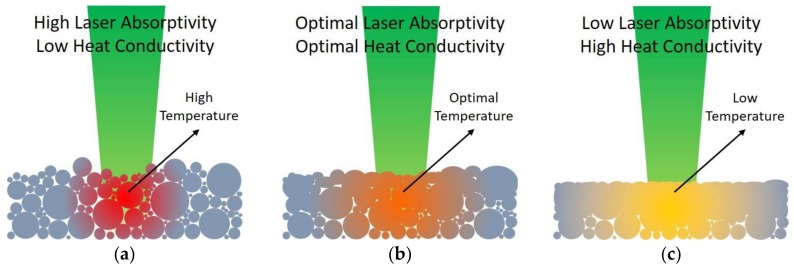
Schematic of the differences in the laser absorptivity and heat conductivity of NiTi materials before and after S1: (**a**) powder bed or poor-melted sample; (**b**) optimized sample; and (**c**) excessively-melted sample.

Among the four samples tested, the measured highest transformation strain was 5.11%, with an average value of 4.61% [31]. This result showed a significant improvement when compared to our previous study of 0.5% transformation strain [30]. Moreover, it was also comparable to the maximum 6% transformation strain of conventionally-produced NiTi [22]. The huge improvement in the transformation strain through producing high-quality samples could be attributed to the differences in the laser absorptivity and heat conductivity of NiTi materials before and after S1 [40,48]. Figure 3 is a revised schematic diagram [31] that illustrates the different states of the material as a result of introducing repetitive scanning.

It is generally well-known that powder materials have much higher laser absorptivity than materials in their dense form [48]. When the energy density directed to the powder bed (Figure 3a) was too high, its poor heat conductivity and excellent laser absorptivity assisted in generating a greater degree of melting during S1. Hence, the samples produced (Figure 3c) had a high density. Consequently, their reduced laser absorptivity decreased the amount of energy absorbed during S2. Moreover, their high heat conductivity aided in the transfer of the absorbed energy. Hence, these high-density samples experienced a lower temperature during S2, leading to a poor wetting condition, generation of porosity, and balling effect [42,49]. 

Conversely, when the energy density inputted was optimal, the samples fabricated (Figure 1a and Figure 3b) were less dense. Thus, they experienced a higher temperature than the denser samples (Figure 3c) during S2. This resulted in the reduction in the viscosity of the molten pool, wetting angle, and surface tension [40,42,44]. Eventually, these occurrences contributed to the production of low porosity SLM NiTi without the formation of the balling effect (Figure 1b). However, in the case of insufficient energy density delivered during S1, a minimum amount of melting will be generated. Hence, the produced parts (Figure 3a) will experience a higher temperature at localized points during S2 due to the retention of high laser absorptivity and low heat conductivity. Subsequently, a more pronounced temperature gradient is produced, resulting in high thermal stresses and warpage of the samples [50].

Nevertheless, despite the improvement in the transformation strain, the presence of the permanent strain indicates the presence of incomplete shape recovery. The proposed future research for further improving the transformation strain can be found in previous work of ours [31].

### 2.4. Applications of SLM NiTi

Among the research done on the applications of SLM NiTi, its biocompatibility effect has been the most widely studied [14]. Shishkovsky et al. reported the absence of free Ni, Ti, and other intermetallic phases such as Ni_3_Ti and NiTi_3_ during the rapid solidification of their samples [13,25]. This indicated that the samples produced were suitable for medical procedures due to the lack of precipitated phase and highly homogeneity in composition. For example, an excessive amount of Ni released can cause toxicity, carcinogenic effects, and immune responses [5]. 

Another research by Habijan et al. worked on the determination of the suitability of SLM NiTi for biomedical implants [14]. They also experimented on their samples to determine if SLM NiTi could be a potential carrier for the human mesenchymal stem cells. According to the obtained results, the surface topography of the samples and the morphology of the cells could be varied by adjusting the SLM parameters. Although both dense and porous samples were deemed to be suitable for carrying human mesenchymal stem cells, porous samples tend to release more Ni ions. Nonetheless, the amount of Ni released was still much lower than the cytotoxic concentration. The mean concentration of Ni ion released could also be significantly reduced by decreasing the laser spot size. Thus, SLM NiTi can be concluded to be a suitable carrier.

In contrast, the NiTi samples were observed to be susceptible to the release of loose particles. This is unacceptable for biomedical implants. However, the number of particles released depends on the orientation and focus adjustment of the laser. Horizontal surfaces should be produced using a less focused laser due to a large but plain laser intensity distribution within the laser-material interaction zone. Vertical surfaces can be produced with a focused laser and a reduced powder layer thickness to minimize the powder particles released. Nevertheless, a smaller powder size is required for decreasing the layer thickness, which may come with an increase in the content of impurities. In addition, smaller powder particles have poor flowability as a result of powder agglomeration. It is also not possible to totally prevent any particle released by the SLM NiTi parts. Hence, post-processing treatments such as an additional sintering process or subjecting the built parts to ultrasonic cleaning for a prolonged period of time may be required. More work is still needed in order to successfully produce SLM NiTi components suited for biomedical implants. A good prospect of SLM NiTi is that the NiTi implants can be easily tailored for individuals. 

Other than biomedical applications, SLM NiTi can also be utilized in other areas. For instance, Clare et al. produced SLM NiTi samples that demonstrated a much gradual phase transformation [18]. This gradual response has presented many potential benefits for the applications in MEMS. For example, the micro-thermal actuators for heat exchangers may require more discrete intervals of actuation. This can be achieved with an SMA that exhibits gradual phase transformation. Unless the temperature of the NiTi material can be controlled precisely within a certain range of phase transformation, an abrupt phase change may only result in a near 100% austenitic composition or martensitic phase. Furthermore, the gradual transformation of microstructural phases also tends to allow better control of the material properties. A complete martensitic composition is softer and more malleable while a 100% austenitic phase is relatively hard with a much higher Young’s modulus [51]. With a gradual phase transformation, the amount of each phase present can be controlled more easily by regulating the temperature. Thus, the material properties will also be easier to manage. The capability to have control over both form and material properties may find many other different applications. Overall, due to the advantage of addressing the poor machinability of NiTi, the SLM process presents an endless possibility of producing complex smart structures for various engineering sectors. Hopefully, in the near future, additional fields of application of SLM NiTi will be reported.

## 3. Summary

In this paper, various studies conducted on the SLM of NiTi were reviewed together with some of the results of our ongoing research. These findings were mainly classified into the effects of energy density, the influence of SLM process parameters, shape memory responses of SLM-produced NiTi, and potential applications of SLM NiTi.

1. Three adverse effects were observed when energy density directed to the powder bed exceeded the optimal amount; the increase in porosity, transformation temperatures, and impurities of SLM NiTi.
2. SLM process parameters have a strong influence on the transformation characteristics, mechanical properties, porosity, and geometrical characteristics of SLM NiTi. Optimization of the parameters is required to obtain a SLM NiTi with controllable transformation characteristics, desired mechanical properties, minimal porosity, low impurity, and optimal shape memory responses.
3. SLM NiTi can demonstrate comparable SME and superelasticity as the conventionally-produced NiTi. However, its shape memory responses differ according to the loading direction. Furthermore, it is possible to produce a SLM NiTi that exhibits a much gradual phase transformation than the conventional NiTi.
4. The potential applications of SLM NiTi include being carriers for human mesenchymal stem cells, biomedical implants, and MEMS; it is not limited to this review only.
5. Repetitive scanning assisted in producing SLM NiTi samples with high-quality. However, the properties of the fabricated parts are dependent on the laser absorptivity and heat conductivity of the materials before and after the first scan. The energy density inputted must be carefully controlled to achieve the desired mechanical and functional properties.

## Figures and Tables

**Table 1:** Various strain readings of the sample tested in Figure 2.

Maximum Strain (%)	Residual Strain (%)	Transformation Strain (%)	Permanent Strain (%)
7.95	6.55	5.11	1.17
